# Assessing the risk of bias of clinical trials with large language models and ROBUST-RCT: a feasibility study

**DOI:** 10.1038/s41598-026-44303-z

**Published:** 2026-03-17

**Authors:** Pedro Rodrigues Vidor, Yohan Casiraghi, Adolfo Moraes de Souza, Maria Inês Schmidt

**Affiliations:** 1 School of Medicine, Universidade Federal do Rio Grande do Sul, Hospital de Clínicas de Porto Alegre, R. Ramiro Barcelos, 2400, Porto Alegre (RS), Porto Alegre, 90035-003 Brazil; 2 Postgraduate Program in Epidemiology, Universidade Federal do Rio Grande do Sul, Hospital de Clínicas de Porto Alegre, Porto Alegre, Brazil

**Keywords:** Risk of bias, Inter-rater reliability, Randomized controlled trials, Large language models, Health care, Mathematics and computing, Medical research

## Abstract

Risk of bias assessment is a crucial step in evidence synthesis. The traditionally adopted tool, however, is complex, resource-intensive, and unreliable. While prior investigations have focused on whether Large Language Models (LLMs) could perform assessments with RoB 2, this study is the first to evaluate the reliability of ROBUST-RCT, a novel risk-of-bias tool, as applied by humans and LLMs. Reviewers working independently used ROBUST-RCT to assess different aspects of a sample of RCTs and then reached a consensus through discussion. A chain-of-thought prompt instructed four LLMs on how to apply ROBUST-RCT. The primary analysis used Gwet’s AC2 to assess inter-rater reliability based on all the final ratings (i.e., the ratings in the second step of the tool) for all the core items of the ROBUST-RCT. A sample of 54 assessments, derived from 9 studies, was compared for each LLM against human consensus. In the primary analysis, Gwet’s AC2 inter-rater reliability varied across the LLMs. DeepSeek-R1, the lowest performer, yielded an AC2 of 0.46 ( 95% CI: 0.24 to 0.69). On the other side, Gemini 2.5 Pro Preview – the model with higher consistency with human consensus – yielded an AC2 of 0.69 (95% CI: 0.54 to 0.84). With 95% confidence, three of the four tested LLMs achieved ‘moderate’ or higher reliability based on benchmarking. LLMs could be helpful in the risk-of-bias assessment of systematic reviews using the ROBUST-RCT tool.

**Supplementary Information:**

The online version contains supplementary material available at 10.1038/s41598-026-44303-z.

## Introduction

Although considered the gold standard of clinical research, randomized controlled trials (RCTs) are not immune to bias. Currently, the risk of bias in RCTs is usually assessed using Cochrane’s Risk of Bias 2 (RoB 2)^1^, which has well-documented limitations: its application is time-consuming^2^, complex even for experienced researchers^3^, and results in low inter-rater reliability^2,4^.

A time-consuming and complex tool, however, may not be the most suitable option for the increasingly large body of evidence and techniques available. The number of published RCTs has been increasing for decades^5^; from 1991 to 2020, it grew at an average annual rate of 7.68%^6^, whereas the number of included studies in systematic reviews of interventions has remained constant^7^. Furthermore, the use of novel and more demanding approaches, such as living systematic reviews and network meta-analysis, has also gained traction in the scientific literature^6,8^.

Aligned with the complexity of methodologies, the median time from protocol registration to publication of a systematic review is 2.2 years^9^. The median publication timeline, based on a 5-year window, has increased from 2000 to 2014 compared with 2015 to 2019^9^. In a previous study, 7% of the reviews were already outdated at the time of publication, and 11% were outdated within 2 years of the systematic search^10^. Taken together with the growing body of evidence, this delay in publication times – along with its potential impact in patient’s health – may indicate the need for faster frameworks.

Given the increasing burden of conducting high-quality reviews, artificial intelligence (AI) could help scientists accelerate research and ease workflows^11^. AI tools have been integrated into scientific research^12^, with some authors reporting substantial advantages^11,13^. Concerns regarding the standardization and reporting of these technologies have been raised^14,15^, underscoring the need for reliable, transparent, and realistic workflows^16^.

The automation of the risk-of-bias assessment, which has been proposed for years^17^, could be feasible by processing scientific texts with large language models (LLMs), a specific type of AI. Aligned with the recent integration of these tools into the health sciences^18–22^, the registration form for the systematic review protocol database PROSPERO^23^ already includes the term “machine” in all fields for reporting the number of people who will assess the risk of bias^24^. However, most of the literature on the use of LLMs for risk-of-bias assessment has focused on RoB 2^20,25,26^.

The introduction of the ROBUST-RCT^27^ provides an alternative to the RoB-2 approach, with several differences in its conception and development. The novel tool has been designed by a panel of epidemiologists and methodologists, aiming to contrast with the complexity of other tools in medical research by balancing ease of use and rigor. The six ROBUST-RCT core items were selected based on criteria such as whether there is evidence for their influence in the effect estimation and the frequency with which the corresponding bias affects RCTs. Its development included a usability test with junior reviewers^27^, emphasizing the goal of being simpler than previous tools.

Therefore, a critical opportunity lies in combining technological advancements with newer instruments for risk-of-bias assessment. This feasibility study is the first to investigate the inter-rater reliability of four LLMs using ROBUST-RCT, compared with human consensus. Furthermore, it provides data, previously unavailable in the scientific literature, on inter-rater reliability across human reviewers applying ROBUST-RCT and across different LLMs.

## Methods

We designed a feasibility study involving humans and LLMs using the ROBUST-RCT tool. A retrospective registration^28^ has been documented in the Open Science Framework.

### Sample

We searched for *(“drug therapy”[MeSH Terms]) AND (Randomized Controlled Trial [Publication Type]) AND (“pubmed pmc”[filter])* on PubMed on May 18, 2025, sorted by the platform’s default algorithm. The choice of articles on PubMed Central enables standardization using the same PDF file. We exported the first 10000 results to a file. We then randomized rows using Google Sheets and selected the first 20 rows in the new order.

The initial sample size was designed to yield, even with a potentially high exclusion rate, more included studies than in a typical systematic review. In cross-sectional descriptive analyses, the median number of included studies in meta-analyses with two or more studies was only 3, with three-quarters having five or fewer studies^29^. The number of included studies per meta-analysis has not increased over time^7^, although the number of RCTs has^6^.

For studies that assess inter-rater reliability using widely adopted tools, a common practice is to conduct secondary analyses of data from existing systematic reviews^2,26,30^. This practice was infeasible for the ROBUST-RCT, as the tool had recently been published. The choice of the “judgment set” as a unit in the primary analysis further enhances the statistical power of this study (see “Primary analysis”), while the method of randomizing included studies aims to ensure a diversified sample of articles.

### Exclusion criteria

The full text of each article reporting an RCT was screened for eligibility by independent reviewers. Three reviewers, working in pairs of independent reviewers, screened the full text for exclusion based on predefined criteria (see Supplementary Table 1). Any disagreements regarding article exclusion were resolved through consensus.

### Human assessment

ROBUST-RCT proposes itself as straightforward enough to be used by junior systematic reviewers, with its application for this subset of researchers being part of the tests originally employed to evaluate its usability in the ROBUST-RCT development paper^27^. Therefore, in our experiment, a group of three early-career researchers, acting as independent reviewers, applied the ROBUST-RCT tool in accordance with the recommendations outlined in the ROBUST-RCT manual.

All of the reviewers involved were medical students who contributed to at least one systematic review and had prior knowledge about Cochrane’s RoB-2, the traditional tool for risk-of-bias assessments. Digital copies of the full manual were used as a guide for the assessment. Degrees represented the diversity of a typical research setting: one was an undergrad, one had a prior Bachelor’s degree, and one had a Master of Science degree. All of them had critical knowledge about epidemiology and health sciences, having completed at least half of medical school. A seasoned epidemiologist was available to discuss any conceptual questions when needed. All the individuals documented their judgments in a standardized spreadsheet.

ROBUST-RCT has 6 items, each with 2 steps, and each step has four possible answers (Supplementary Table 2), except for step 1 of item 6. The value ranges for item 6, step 2 (Supplementary Table 3), followed the thresholds specified in the example in the ROBUST-RCT manual. Assessment was performed based exclusively on the article. There was an explicit recommendation to disregard other external sources of information—such as study protocols—to preserve the fairness of the assessment conditions between humans and AIs. Disagreements between assessors were resolved in a consensus meeting.

To ensure standardization in the application of ROBUST-RCT, only the primary outcome of each study was assessed. The original ROBUST-RCT spreadsheet was used, with the “outcome” field used to describe each study’s primary outcome. This approach enabled a swift operational workflow by allowing files to be analyzed in the same order and format as originally used by the reviewer, thereby avoiding the need to adjust the dataset due to potential inconsistencies in reporting. By simplifying the framework, this study also aimed to mitigate the risk of human errors in the data curation. Articles that had been excluded by consensus were not subjected to evaluation using ROBUST-RCT. All multiple-choice fields were mandatory.

### AI assessment

A standardized prompt, based on the ROBUST-RCT manual^27^, instructed the LLMs via their web graphical interfaces to perform the assessment using the PDF file available for download from PubMed Central. The input uses the Chain-of-Thought (CoT) strategy^31–33^. Its format includes an initial contextualization, a step-by-step guide on ROBUST-RCT, and an output format. The full CoT prompt is available in Appendix 1.

This choice of CoT format, which specifies how to apply the risk-of-bias tools, also derives from the aim of enabling LLMs to use a more recent tool. Compared with the ROBUST-RCT, traditional alternatives that have been available for longer^1,34^ and have been used in numerous publications^35^. Describing the ROBUST-RCT tool could, therefore, contribute to ensuring that the LLMs were not compelled to use other criteria (e.g., from Cochrane’s RoB-2).

This prompt was submitted with each study’s article—and, when necessary, supplemental materials. We tested four LLMs: GPT-4-turbo (OpenAI) on May 31, 2025; Gemini 2.5 Pro Preview (Google) on May 30, 2025; DeepSeek-R1 (Hangzhou DeepSeek Artificial Intelligence) on May 31, 2025; and Qwen3-235B-A22B (Alibaba Cloud) on May 31, 2025. The last two models are open-source^36,37^.

Given the complexity of the task, advanced reasoning tools^38,39^ – a capability of the LLM to make inferences about its sources before sending the final output – were activated when available. The option “Think for longer” for GPT-4-turbo; the option “Thinking” for Qwen3-235B-A22B. Other LLMs – DeepSeek-R1 and Gemini 2.5 Pro Preview – did not need activation of this tool because reasoning was default, in contrast with their simpler and faster models – DeepSeek-V3 and Gemini 2.5 Flash Preview.

### Statistics

Data from human reviewers were filtered into a final dataset, which included only those articles submitted for LLM evaluation. The inter-rater reliability analysis between humans and LLMs used only the final consensus ratings from the human reviewers. Statistical analyses were all performed in the R language version 4.4.0. The analysis scripts used the irrCAC package^40^. A validation step, which ensures the accuracy and quality of the analysis script, was performed by successfully replicating results from prior papers^4,41^ using a publicly available dataset^42^.

### Primary analysis

We established a set of assessments for the main analysis that included all final judgments (steps 2 of ROBUST-RCT, core items 1-6), which we defined from now on as the “judgment set”. This choice is based on clinical relevance and its contribution to statistical power. From a medical perspective, this set combines the most critical steps of the assessments across multiple aspects of RCTs. In data analysis, aggregating these data also enhances precision and stability.

The primary analysis is a Gwet’s AC2 inter-rater reliability coefficient^43^ of the judgment set, divided into four distinct analyses comparing human consensus against each of the 4 LLMs. The calculation for this primary analysis employed the following parameters: ordinal weights, 4 possible levels, and a 95% confidence level.

This measurement aims to enhance the robustness of the inter-rater reliability analysis. Gwet’s AC2 does not suffer from the same issues as Kappa coefficients^44–49^, which are strongly affected by the frequency distribution^47^, biased in real-world scenarios^46^, and may present paradoxical results^44,45^. The choice of parameters, based on those previously used for similar data^41^, considers the magnitude of agreement. Thus, distant divergences (e.g., “Definitely Low” and “Definitely High”) are penalized more heavily than close divergences (e.g., “Probably Low” and “Probably High”).

This analysis incorporates a probabilistic benchmark^43^ in conjunction with the Landis and Koch classification^50^, which considers not only the coefficient but also its standard error (SE). This is necessary due to the differences in precision for the same coefficient (e.g., 0.41) with different SEs (e.g., 0.03 and 0.35), which would limit the interpretation of the results. This problem is mitigated by a process where the probabilities of a coefficient falling into each value range or a higher one are computed by calculating the interval membership probability (IMP). Thus, Gwet’s benchmark allows inferring the minimum classification to which that coefficient belongs with 95% confidence.

### Exploratory analyses

As a hypothesis-generating study, the primary analysis – which represents its initial and predefined aim – was complemented by a range of additional analyses. Three exploratory analyses were performed. The first and third analyses were predefined but were not the main aim of the study; the second analysis, however, was designed only after the data were available.

1. Fleiss’ Kappa^51^ of the “judgment set” among different human assessors before consensus, using the following parameters: “identity” weight, 4 levels, and 95% confidence. The coefficients were interpreted using the classification scheme developed by Landis and Koch. This approach aims to provide comparability to previous studies of human inter-rater reliability in risk-of-bias tools.
2. Analysis of the direction of bias for each LLM’s assessments relative to human consensus for the judgment set. To quantify divergences, numerical values from 0 (definitely yes, definitely low) to 3 (definitely no, definitely high) were assigned to each possible response level for each ROBUST-RCT item (Appendix 3). We then performed a paired Wilcoxon signed-rank test and adjusted p-values with the Bonferroni correction.
3. Assessment of inter-rater reliability using Gwet’s AC2, applied to each multiple-choice step individually, using the same parameters as the primary analysis.

## Results

From our initial database of 20 randomly selected RCTs, 11 were excluded based on the previously mentioned criteria, resulting in 9 articles for further analysis. None of the included studies (Supplementary Table 4) had been previously assessed with ROBUST-RCT. Appendix 2 documents all LLM outputs. Raw data and scripts are publicly available^52^.

As the judgment set includes step 2 assessments from 6 different core items for 9 RCTs, the sample size for the primary analysis had a total of 54 comparisons of evaluations between LLMs and human consensus. Step-level exploratory analysis has a smaller sample size (n = 9) because it focuses on each multiple-choice step of ROBUST-RCT individually.

### Primary analysis

**Fig. 1:**
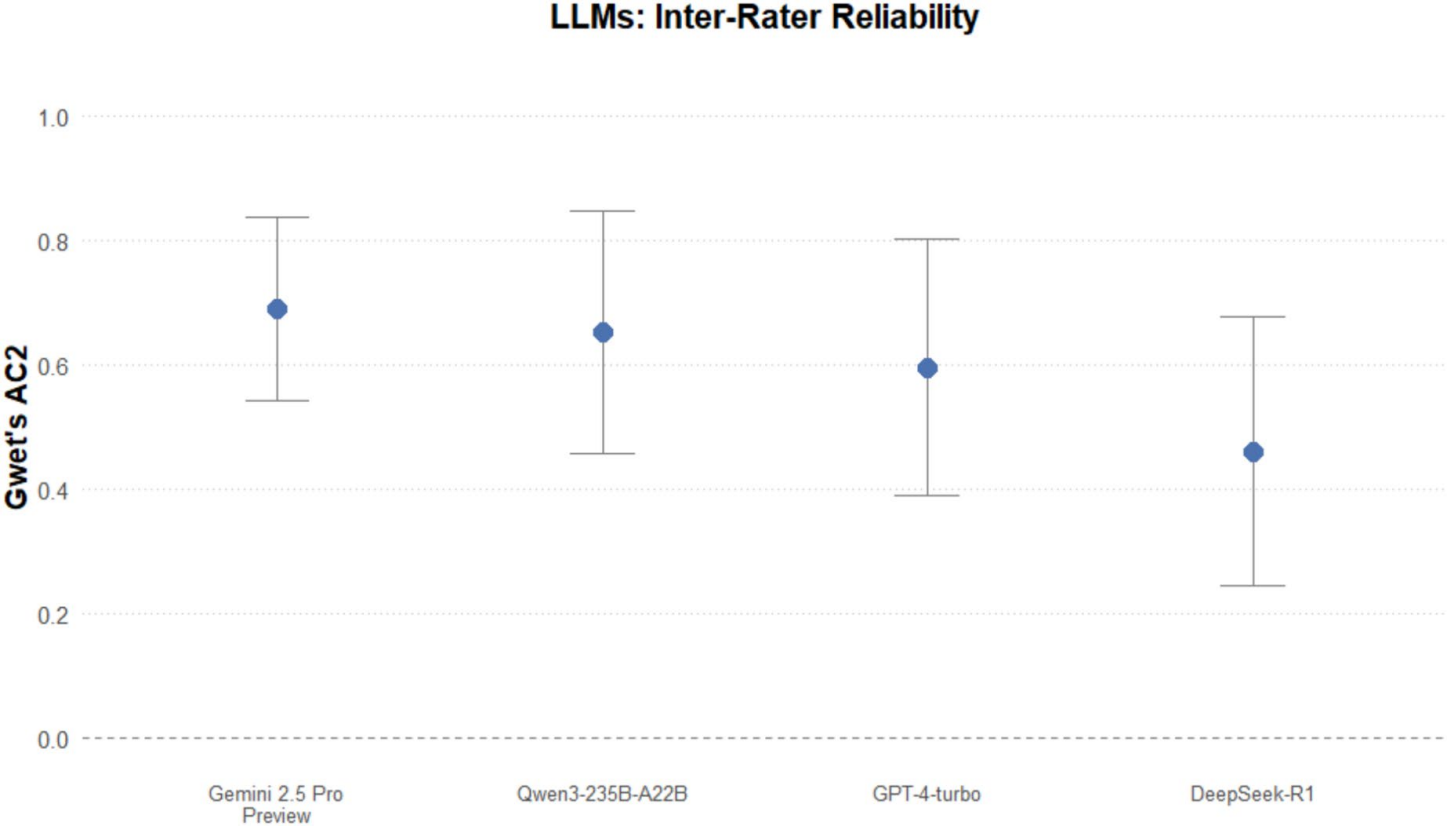
Gwet’s AC2 for the judgment set between human consensus and each LLM.

The analysis of the judgment set resulted in the following Gwet’s AC2 coefficients (Figure 1) between human consensus and the LLMs (Table 1) and the following benchmarks (Supplementary Table 5).

**Table 1:** Gwet’s AC2 for the judgment set. For inter-rater reliability measures, percent agreement denotes the extent to which the raters agree, and percent expected denotes the percentage of potential agreement expected by chance alone. Both metrics incorporate the weighting matrix assigned to the categories. For a Gwet’s AC2 with ordinal weights and four categories, those weights could be approximately as 0.00, 0.50, 0.83, and 1.00, from the least coherent to the most coherent ratings. Formulas and further details are provided in prior references^40,43,55^.

Comparison	Gwet’s AC2	Percent Agreement	Percent Expected	Standard Error	95% Confidence Interval
GPT-4-turbo	0.60	0.84	0.35	0.11	0.39, 0.81
Gemini 2.5 Pro Preview	0.69	0.87	0.37	0.08	0.54, 0.84
DeepSeek-R1	0.46	0.80	0.32	0.11	0.24, 0.69
Qwen3-235B-A22B	0.65	0.85	0.38	0.10	0.45, 0.85

The probabilistic benchmarking indicates that, with 95% confidence, the AC2 coefficients fall into the following categories: moderate or higher (human consensus vs. GPT-4-turbo, human consensus vs. Gemini 2.5 Pro Preview, human consensus vs. Qwen3-235B-A22B) and fair or higher (human consensus vs. DeepSeek-R1).

### Exploratory analyses

The results of the exploratory analyses described in the methods section are reported below.

#### Fleiss’ Kappa of the judgment set (different humans)

The Fleiss’ Kappa was 0.49 (95% CI: 0.30 – 0.68; Supplementary Table 6) between different assessors before consensus, which falls into the “moderate” category of Landis and Koch.

#### Direction of bias analysis (LLMs compared to human consensus)

By assigning ordinal values to the ROBUST-RCT assessments, it was possible to determine whether there was a statistically significant difference between the LLM assessments and human consensus. Mean values and standard deviations are documented in Supplementary Table 7. Wilcoxon signed-rank tests (Supplementary Table 8) revealed significance in the DeepSeek-R1 assessments compared to human consensus (p-value = 0.0050; Bonferroni-adjusted p-value = 0.0201).

#### Step-level inter-rater reliability (LLMs compared to human consensus)

**Fig. 2:**
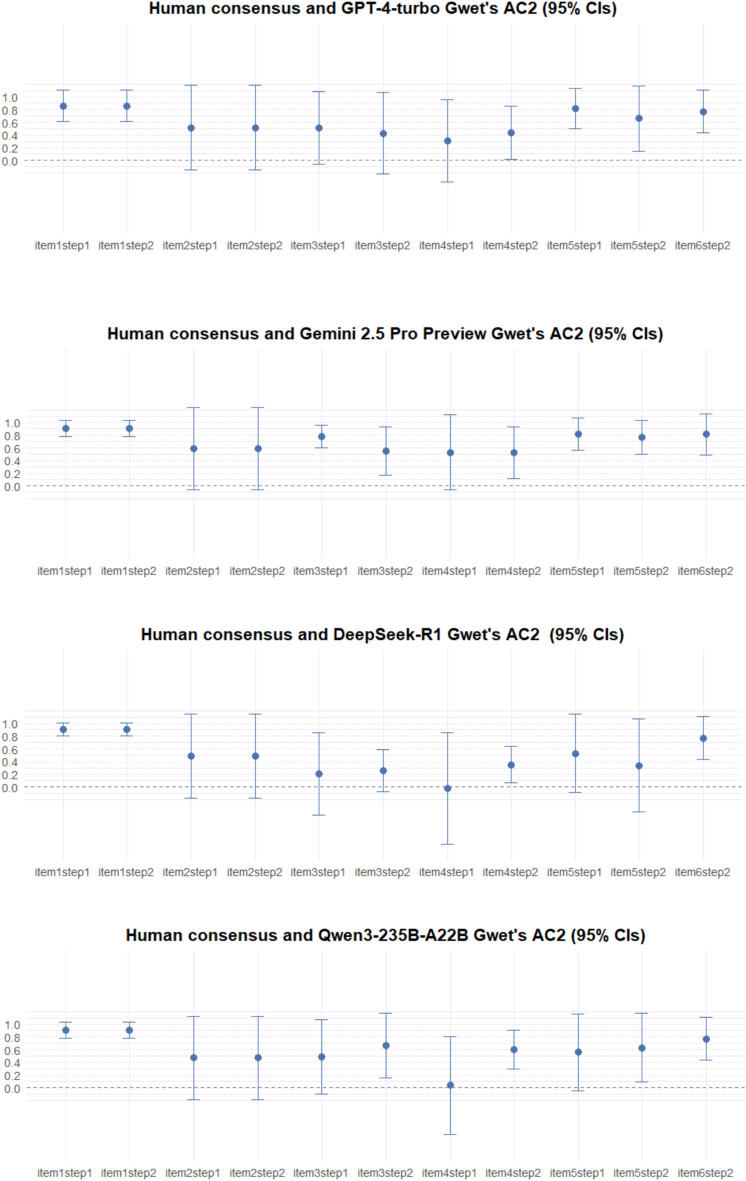
Inter-rater reliability of human consensus and the four tested LLMs: step-level results.

Given that this specific analysis has a smaller sample size, the confidence interval (CI) crossed the null line at least once for each LLM. It occurred 7 times for GPT-4-turbo, 3 for Gemini 2.5 Pro Preview, 5 for DeepSeek-R1, and 5 for Qwen3-235B-A22B. Results are provided in Figure 2. Appendix 4 details all the benchmarks.

## Conclusions

This study suggests that LLMs could help systematic reviewers using the ROBUST-RCT tool. The methodology was partially predefined, although no pre-registration was employed. A key reason was the expectation that framework changes would be required, as the research was conducted shortly after the publication of the ROBUST-RCT (March, 25, 2025)^27^. Human assessments – including consensus – were performed until May 26, 2025; AI assessments were performed between May 30 and 31, 2025.

The research team was prepared for the possibility that the AIs would have issues with the prompting technique, such as failing to comply with instructions and producing outputs that diverged from the required ones. In this case, the prompt, despite adhering to several best practices available at the time of its writing^53,54^, would need to be rewritten. Another concern was whether the reviewers would be able to adequately apply the tool based only on its manual, given the lack of training available at the time.

None of these concerns materialized, which may be due in part to the tool’s design and in part to advances in LLMs. Because three of four tested LLMs demonstrated at least moderate reliability, the findings suggest that integrating these technologies could be incorporated into future workflows for ROBUST-RCT risk-of-bias assessment. The rapid changes in AI technologies should also be considered: newer models may be able to apply the ROBUST-RCT directly from the manual rather than from a summarized instruction in the prompt.

Despite being designed with a small sample, the study achieved the goals of this feasibility stage. Our data suggest that it is feasible to integrate LLMs into risk-of-bias assessments using the ROBUST-RCT tool, a topic that has not been previously addressed in the literature. Therefore, rather than a limitation, this sample size may serve as a basis for further, more extensive studies and also demonstrates robust approaches to measuring inter-rater reliability, from which future research on risk-of-bias could benefit.

The roles of LLMs in evidence synthesis remain to be defined. For risk-of-bias assessments, it could be used as a tier-breaker, serving as a third reviewer alongside two human reviewers, or to identify potential errors. Another alternative would be to automate evaluations and then assign human researchers to judge complex cases and disagreements. Semi-automated workflows could be valuable in reducing the burden on research teams, enabling faster updates to living systematic reviews, and allowing guideline discussions to focus more on the findings than on risk-of-bias assessments.

The robustness of our results was assessed through rigorous benchmarking and interpretation. As demonstrated in our analysis, even a coefficient that might be directly interpreted as “substantial” — such as for Gemini 2.5 Pro Preview (Gwet’s AC2=0.69) — is conservatively classified as “moderate” or higher with 95% confidence. It should also be noted that using other prompt formats, such as the Reflection of Thoughts^33^, may yield different and potentially better results.

**Fig. 3:**
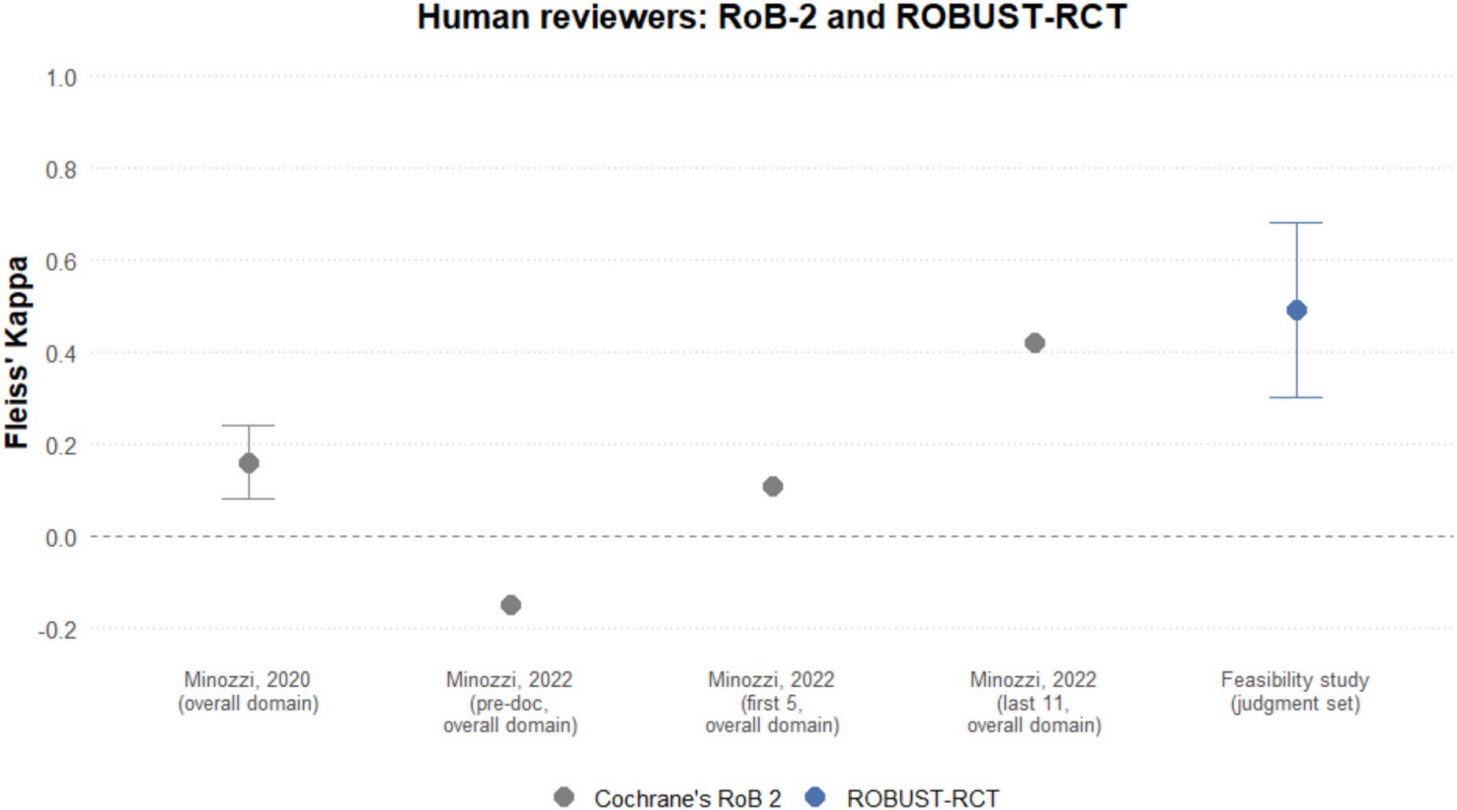
Inter-rater reliability of human reviewers using the Cochrane’s RoB-2 and the ROBUST-RCT tool. One of the previous papers^2^ on this subject did not report its confidence interval.

Our inter-rater reliability (0.49 [95% CI: 0.30 – 0.68]) for humans using ROBUST-RCT is comparable to the reported measures of Fleiss’ Kappa for the RoB 2 tool (Figure 3). A prior study^4^ found 0.16 (95% CI: 0.08-0.24) for the RoB 2 “overall” domain, classified as “slight”. Another study^2^ assessed inter-rater reliability with varying degrees of instruction. Its results for the “overall” judgment ranged from “no agreement” (-0.15) before the use of an implementation document, to “moderate” (0.42) for the last 11 studies evaluated – no CI was reported in that latter study.

The employment of junior scientists in our feasibility study, rather than seasoned experts, also suggests consistency of risk-of-bias evaluations with ROBUST-RCT. The inter-rater reliability measure, however, must be interpreted cautiously. Not only is this an exploratory analysis, but other studies with similar measures had low sample sizes. A reliable measure would require a larger sample with greater statistical power and a prespecified protocol.

The use of Fleiss’ Kappa, despite being the standard in previous reports, also has potential limitations, as noted previously (see Methods). It should be noted that higher interrater reliability, although desirable, does not necessarily imply improved detection of bias; assessing construct validity would require a different study design. Additionally, the sample size of this work leads to wide confidence intervals. Thus, the classification based on Landis and Koch’s^50^ thresholds is limited by the potential instability of the results.

Nevertheless, it should be emphasized that a deterministic view — limited to the usual arbitrary Landis and Koch classifications without benchmarking — contrasts with the methodological rigor required for medical sciences. Our work aligns with prior publications^41,43,55^, thereby reinforcing the need for robustness in inter-rater coefficient calculation and benchmarking processes in study design.

The direction of bias analysis indicated that the worst-performing LLM – DeepSeek-R1 – may have been more stringent in its evaluations than its human counterparts. DeepSeek-R1’s lack of multimodality may also have contributed to its inferior reliability. While most LLMs have some sort of vision, DeepSeek-R1 just converts submitted PDFs into plain text through optical character recognition. As a result, this process could lead to the loss of information from figures, tables, and diagrams.

Combined with the higher mean value of DeepSeek-R1 (Supplementary Table 6), the Wilcoxon signed-rank test suggests a more stringent evaluation than human consensus. In conjunction with the primary analysis (Gwet’s AC2), this finding suggests that the current version of DeepSeek-R1 may be inadequate due to the presence of alternatives that yield more consistent assessment results. Moreover, as stated before (see ‘Methods’), this analysis is an expansion of the initial scope of this study.

The results, and particularly the direction of bias, should be interpreted with caution, as our approach assumes that human consensus is the gold standard. It is plausible that, in some instances of disagreement, the AI’s assessment was more accurate. This potential inversion, with LLMs performing assessments more reliably than humans, may become more prevalent as models improve^56^. Conversely, there is a growing debate that relying on AIs could be ‘deskilling’ physicians, a claim supported by at least one piece of evidence in medical science^57^. It is also hypothetically possible that, in some instances, both humans and LLMs could make the same mistakes.

The last of the exploratory analyses—a Gwet’s AC2 for each multiple-choice step of each item—showed variation in results at the step level, indicating areas for potential improvements in future and larger studies. There is also the possibility that human training may place greater emphasis on the aforementioned steps, which are associated with lower inter-rater reliability, as human assessment is prone to error. As this analysis used a smaller sample size than the primary analysis, its limited statistical power should be considered.

In contrast to the increasingly explored potential applications of LLMs, several ethical concerns remain largely unanswered. There is now compelling evidence that some models were likely trained on unauthorized data^58^, a practice that – we speculate – could also include scientific publications. As medical scientists increasingly adopt LLMs in their routine practice, it is important to weigh the potential benefits for patient health while acknowledging the risks of AI-related mispractices, including privacy, data security, and copyright issues.

While the medical profession is being redefined by technology, evidence synthesis is also undergoing rapid change. Overall, alongside other investigations of LLMs in health sciences, this study suggests that assessing risk of bias with the ROBUST-RCT may be a future option. Nevertheless, medical scientists should carefully consider how they use AI, taking into account their responsibilities to patients and society.

## Limitations

The sample size limits the precision of the analyses. The small sample size may affect the results (e.g., if, by chance, only well-written articles were rated); inter-rater reliability, therefore, could be affected by resampling (e.g., with more complex articles being randomized for assessment). Focusing on drug interventions could limit the generalization to other interventions.

The standard PubMed search algorithm could bias sampling by favouring studies with particular characteristics (e.g., better reporting could be associated with greater perceived relevance, since the platform’s search weighting is not disclosed). Randomization of studies may partially mitigate this bias (especially compared with approaches that use samples from a single review), but selecting the first 10,000 entries is insufficient to completely eliminate this potential bias, since not all available studies were included in the database before randomization.

The exclusive use of the primary outcome may not reflect the use of ROBUST-RCT in reviews investigating more than one endpoint.

The use of a long CoT-format prompt could be inadequate for models with fewer parameters (e.g., due to a lack of capacity to process all the required information), while also hindering models with stronger inference capabilities and greater context windows (e.g., by standardizing a criterion and not considering its potential exceptions). Future use of LLMs should consider the technique’s adequacy in light of advances in AI research.

The use of LLMs in web interfaces restricts users’ ability to configure the model. The browser version of these tools did not provide options to set the temperature to 0, specify a seed number, or define other parameters. Thus, the inability to configure these options, along with the sample size, may limit the replicability of this work. Moreover, AI companies are often not transparent about model training methodologies, data sources, and weights. For non-open-source LLMs, legacy versions may not be available for use in the future. Fully customizable, locally run open-source models could be considered to enhance the replicability of outputs in future studies.

Some of the LLMs tested were not open-source and were subject to updates and modifications by their providers. The results of these private-sector LLMs are particularly susceptible to model drift. The reliability of all the tested models – including open-source alternatives – is also limited by the technology’s non-deterministic nature.

The reviewers’ instructional level and non-native English proficiency may have affected inter-rater reliability. We explicitly instructed reviewers to avoid using LLMs and maintain blinding for an independent assessment; however, no active barriers were implemented for data storage access or website blocking. Qualitative analyses were beyond the scope of this study and should be considered in future research.

The use of Fleiss’ Kappa to assess the IRR across different humans is limited by inherent methodological issues, including the so-called Kappa paradox and other biases.

Inter-rater reliability is based on consistency between raters and may not accurately reflect the correctness rate. In some instances within this work, human ratings were used as the reference. However, human assessments are also prone to errors and may have failed in some ratings.

## Supplementary Information

Supplementary Information 1.

Supplementary Information 2.

Supplementary Information 3.

Supplementary Information 4.

Supplementary Information 5.

Supplementary Information 6.

Supplementary Information 7.

Supplementary Information 8.

Supplementary Information 9.

Supplementary Information 10.

Supplementary Information 11.

Supplementary Information 12.

## Data Availability

Raw data and scripts available at Open Science Framework: [https://osf.io/crn64/].
