# Correction: Assessing the risk of bias of clinical trials with large language models and ROBUST-RCT: a feasibility study

**DOI:** 10.1038/s41598-026-57081-5

**Published:** 2026-06-24

**Authors:** Pedro Rodrigues Vidor, Yohan Casiraghi, Adolfo Moraes de Souza, Maria Inês Schmidt

**Affiliations:** 1 School of Medicine, Universidade Federal do Rio Grande do Sul, Hospital de Clínicas de Porto Alegre, R. Ramiro Barcelos, 2400, Porto Alegre (RS), Porto Alegre, 90035-003 Brazil; 2 Postgraduate Program in Epidemiology, Universidade Federal do Rio Grande do Sul, Hospital de Clínicas de Porto Alegre, Porto Alegre, Brazil

Correction to: *Scientific Reports* 10.1038/s41598-026-44303-z, published online 17 March 2026

The original version of this Article contained an error in the Abstract.

“A sample of 56 assessments, derived from 9 studies, was compared for each LLM against human consensus.”

now reads:

“A sample of 54 assessments, derived from 9 studies, was compared for each LLM against human consensus.”

The original Article has been corrected.

