# Supplementary material for: Assessing the risk of bias of clinical trials with large language models and ROBUST-RCT: a feasibility study
Source: Sci Rep. 2026 Mar 17;16:13723. doi: 10.1038/s41598-026-44303-z (PMC13125330; doi:10.1038/s41598-026-44303-z)
Supplement: Supplementary file 3 — Supplementary Information 3. [file 41598_2026_44303_MOESM3_ESM.docx]

**Direction of bias: assigned values.**

| pmc_id | question_id | h_consensus | gpt | gemini | deepseek | qwen |
| --- | --- | --- | --- | --- | --- | --- |
| PMC10938176 | item1step1 | 1 | 1 | 0 | 0 | 0 |
| PMC10938176 | item1step2 | 1 | 1 | 0 | 0 | 0 |
| PMC10938176 | item2step1 | 2 | 2 | 2 | 2 | 0 |
| PMC10938176 | item2step2 | 2 | 2 | 2 | 2 | 0 |
| PMC10938176 | item3step1 | 3 | 3 | 3 | 3 | 3 |
| PMC10938176 | item3step2 | 1 | 1 | 1 | 1 | 1 |
| PMC10938176 | item4step1 | 0 | 0 | 0 | 3 | 3 |
| PMC10938176 | item4step2 | 0 | 0 | 0 | 2 | 1 |
| PMC10938176 | item5step1 | 0 | 0 | 0 | 0 | 0 |
| PMC10938176 | item5step2 | 0 | 0 | 0 | 0 | 0 |
| PMC10938176 | item6step2 | 3 | 1 | 1 | 1 | 1 |
| PMC1388094 | item1step1 | 0 | 0 | 0 | 0 | 0 |
| PMC1388094 | item1step2 | 0 | 0 | 0 | 0 | 0 |
| PMC1388094 | item2step1 | 0 | 0 | 0 | 0 | 0 |
| PMC1388094 | item2step2 | 0 | 0 | 0 | 0 | 0 |
| PMC1388094 | item3step1 | 1 | 3 | 2 | 2 | 2 |
| PMC1388094 | item3step2 | 0 | 2 | 2 | 1 | 1 |
| PMC1388094 | item4step1 | 0 | 3 | 3 | 3 | 3 |
| PMC1388094 | item4step2 | 0 | 2 | 2 | 1 | 1 |
| PMC1388094 | item5step1 | 0 | 0 | 0 | 0 | 0 |
| PMC1388094 | item5step2 | 0 | 0 | 1 | 0 | 0 |
| PMC1388094 | item6step2 | 0 | 0 | 0 | 0 | 0 |
| PMC1739189 | item1step1 | 0 | 0 | 0 | 0 | 0 |
| PMC1739189 | item1step2 | 0 | 0 | 0 | 0 | 0 |
| PMC1739189 | item2step1 | 1 | 2 | 1 | 2 | 1 |
| PMC1739189 | item2step2 | 1 | 2 | 1 | 2 | 1 |
| PMC1739189 | item3step1 | 0 | 0 | 1 | 1 | 0 |
| PMC1739189 | item3step2 | 0 | 0 | 1 | 2 | 0 |
| PMC1739189 | item4step1 | 1 | 2 | 1 | 2 | 2 |
| PMC1739189 | item4step2 | 1 | 2 | 1 | 2 | 1 |
| PMC1739189 | item5step1 | 0 | 0 | 0 | 0 | 0 |
| PMC1739189 | item5step2 | 0 | 0 | 0 | 2 | 0 |
| PMC1739189 | item6step2 | 0 | 0 | 0 | 0 | 0 |
| PMC2744681 | item1step1 | 0 | 0 | 0 | 0 | 0 |
| PMC2744681 | item1step2 | 0 | 0 | 0 | 0 | 0 |
| PMC2744681 | item2step1 | 2 | 2 | 2 | 2 | 2 |
| PMC2744681 | item2step2 | 2 | 2 | 2 | 2 | 2 |
| PMC2744681 | item3step1 | 2 | 3 | 2 | 3 | 3 |
| PMC2744681 | item3step2 | 0 | 1 | 2 | 2 | 1 |
| PMC2744681 | item4step1 | 1 | 2 | 2 | 2 | 0 |
| PMC2744681 | item4step2 | 0 | 1 | 2 | 2 | 0 |
| PMC2744681 | item5step1 | 0 | 0 | 1 | 0 | 0 |
| PMC2744681 | item5step2 | 0 | 1 | 1 | 0 | 0 |
| PMC2744681 | item6step2 | 1 | 1 | 1 | 1 | 1 |
| PMC2751593 | item1step1 | 0 | 0 | 0 | 0 | 0 |
| PMC2751593 | item1step2 | 0 | 0 | 0 | 0 | 0 |
| PMC2751593 | item2step1 | 0 | 1 | 1 | 1 | 0 |
| PMC2751593 | item2step2 | 0 | 1 | 1 | 1 | 0 |
| PMC2751593 | item3step1 | 0 | 0 | 0 | 2 | 1 |
| PMC2751593 | item3step2 | 1 | 0 | 0 | 2 | 1 |
| PMC2751593 | item4step1 | 1 | 0 | 0 | 2 | 1 |
| PMC2751593 | item4step2 | 1 | 0 | 0 | 2 | 0 |
| PMC2751593 | item5step1 | 0 | 0 | 0 | 3 | 1 |
| PMC2751593 | item5step2 | 0 | 0 | 0 | 3 | 0 |
| PMC2751593 | item6step2 | 3 | 3 | 3 | 3 | 3 |
| PMC4156368 | item1step1 | 0 | 0 | 0 | 1 | 0 |
| PMC4156368 | item1step2 | 0 | 0 | 0 | 1 | 0 |
| PMC4156368 | item2step1 | 3 | 0 | 0 | 0 | 0 |
| PMC4156368 | item2step2 | 3 | 0 | 0 | 0 | 0 |
| PMC4156368 | item3step1 | 3 | 3 | 3 | 3 | 3 |
| PMC4156368 | item3step2 | 3 | 1 | 2 | 2 | 3 |
| PMC4156368 | item4step1 | 3 | 3 | 3 | 3 | 3 |
| PMC4156368 | item4step2 | 3 | 2 | 2 | 2 | 2 |
| PMC4156368 | item5step1 | 3 | 1 | 1 | 0 | 0 |
| PMC4156368 | item5step2 | 3 | 1 | 1 | 1 | 0 |
| PMC4156368 | item6step2 | 0 | 0 | 0 | 0 | 0 |
| PMC4282380 | item1step1 | 1 | 3 | 2 | 1 | 2 |
| PMC4282380 | item1step2 | 1 | 3 | 2 | 1 | 2 |
| PMC4282380 | item2step1 | 2 | 3 | 1 | 1 | 1 |
| PMC4282380 | item2step2 | 2 | 3 | 1 | 1 | 1 |
| PMC4282380 | item3step1 | 0 | 3 | 1 | 3 | 3 |
| PMC4282380 | item3step2 | 0 | 3 | 1 | 2 | 3 |
| PMC4282380 | item4step1 | 1 | 3 | 1 | 3 | 3 |
| PMC4282380 | item4step2 | 1 | 3 | 1 | 2 | 3 |
| PMC4282380 | item5step1 | 0 | 2 | 1 | 2 | 3 |
| PMC4282380 | item5step2 | 0 | 3 | 1 | 3 | 3 |
| PMC4282380 | item6step2 | 2 | 3 | 2 | 3 | 3 |
| PMC5651318 | item1step1 | 1 | 1 | 1 | 1 | 1 |
| PMC5651318 | item1step2 | 1 | 1 | 1 | 1 | 1 |
| PMC5651318 | item2step1 | 1 | 1 | 1 | 1 | 2 |
| PMC5651318 | item2step2 | 1 | 1 | 1 | 1 | 2 |
| PMC5651318 | item3step1 | 1 | 0 | 0 | 0 | 0 |
| PMC5651318 | item3step2 | 0 | 0 | 0 | 0 | 0 |
| PMC5651318 | item4step1 | 1 | 1 | 0 | 0 | 0 |
| PMC5651318 | item4step2 | 1 | 1 | 0 | 0 | 0 |
| PMC5651318 | item5step1 | 1 | 1 | 0 | 1 | 2 |
| PMC5651318 | item5step2 | 1 | 1 | 0 | 0 | 2 |
| PMC5651318 | item6step2 | 0 | 0 | 0 | 0 | 0 |
| PMC5820149 | item1step1 | 1 | 0 | 0 | 0 | 0 |
| PMC5820149 | item1step2 | 1 | 0 | 0 | 0 | 0 |
| PMC5820149 | item2step1 | 0 | 0 | 0 | 1 | 0 |
| PMC5820149 | item2step2 | 0 | 0 | 0 | 1 | 0 |
| PMC5820149 | item3step1 | 0 | 0 | 0 | 1 | 0 |
| PMC5820149 | item3step2 | 0 | 0 | 0 | 2 | 0 |
| PMC5820149 | item4step1 | 1 | 0 | 0 | 1 | 0 |
| PMC5820149 | item4step2 | 1 | 0 | 0 | 2 | 0 |
| PMC5820149 | item5step1 | 0 | 0 | 0 | 0 | 0 |
| PMC5820149 | item5step2 | 0 | 0 | 0 | 0 | 0 |
| PMC5820149 | item6step2 | 1 | 0 | 0 | 0 | 0 |
