# Supplementary material for: Assessing the risk of bias of clinical trials with large language models and ROBUST-RCT: a feasibility study
Source: Sci Rep. 2026 Mar 17;16:13723. doi: 10.1038/s41598-026-44303-z (PMC13125330; doi:10.1038/s41598-026-44303-z)
Supplement: Supplementary file 4 — Supplementary Information 4. [file 41598_2026_44303_MOESM4_ESM.docx]

**Exploratory analyses: step-level Gwet’s AC2**

**COEFFICIENTS**

**GPT-4-turbo**

dimension method coefficient stderr p_value

item1step1 Gwet_AC2 0.86033 0.12542 0.0001297287304

item1step2 Gwet_AC2 0.86033 0.12542 0.0001297287304

item2step1 Gwet_AC2 0.51497 0.33985 0.168162862

item2step2 Gwet_AC2 0.51497 0.33985 0.168162862

item3step1 Gwet_AC2 0.51171 0.29082 0.1165224064

item3step2 Gwet_AC2 0.42531 0.32639 0.2287993914

item4step1 Gwet_AC2 0.31075 0.32991 0.3738033851

item4step2 Gwet_AC2 0.43509 0.21524 0.07788591542

item5step1 Gwet_AC2 0.8125 0.16195 0.001030767486

item5step2 Gwet_AC2 0.66165 0.26104 0.03499717444

item6step2 Gwet_AC2 0.77186 0.17009 0.001904500295

**Gemini 2.5 Pro Preview**

dimension method coefficient stderr p_value

item1step1 Gwet_AC2 0.90155 0.0637 6.04E-07

item1step2 Gwet_AC2 0.90155 0.0637 6.04E-07

item2step1 Gwet_AC2 0.58916 0.33044 0.1124416747

item2step2 Gwet_AC2 0.58916 0.33044 0.1124416747

item3step1 Gwet_AC2 0.78082 0.09239 2.94E-05

item3step2 Gwet_AC2 0.55 0.1946 0.02227546613

item4step1 Gwet_AC2 0.53064 0.29802 0.1128515439

item4step2 Gwet_AC2 0.52381 0.20822 0.03605197786

item5step1 Gwet_AC2 0.81772 0.12804 0.0002121238825

item5step2 Gwet_AC2 0.77028 0.13681 0.0004924805461

item6step2 Gwet_AC2 0.81226 0.16414 0.001122726099

**DeepSeek-R1**

dimension method coefficient stderr p_value

item1step1 Gwet_AC2 0.90155 0.05211 1.27E-07

item1step2 Gwet_AC2 0.90155 0.05211 1.27E-07

item2step1 Gwet_AC2 0.48645 0.33617 0.185907204

item2step2 Gwet_AC2 0.48645 0.33617 0.185907204

item3step1 Gwet_AC2 0.2063 0.32949 0.5486799277

item3step2 Gwet_AC2 0.25311 0.16879 0.1721071345

item4step1 Gwet_AC2 -0.02395 0.44824 0.9586949521

item4step2 Gwet_AC2 0.3527 0.14718 0.04342671427

item5step1 Gwet_AC2 0.52673 0.31483 0.1328470942

item5step2 Gwet_AC2 0.33657 0.37182 0.3918065584

item6step2 Gwet_AC2 0.77186 0.17009 0.001904500295

**Qwen3-235B-A22B**

dimension method coefficient stderr p_value

item1step1 Gwet_AC2 0.90155 0.0637 6.04E-07

item1step2 Gwet_AC2 0.90155 0.0637 6.04E-07

item2step1 Gwet_AC2 0.46846 0.33257 0.1966126259

item2step2 Gwet_AC2 0.46846 0.33257 0.1966126259

item3step1 Gwet_AC2 0.48645 0.29821 0.1414846238

item3step2 Gwet_AC2 0.66316 0.26148 0.034920893

item4step1 Gwet_AC2 0.03933 0.39247 0.9226501629

item4step2 Gwet_AC2 0.60049 0.15264 0.004332687702

item5step1 Gwet_AC2 0.55828 0.30565 0.1051942381

item5step2 Gwet_AC2 0.63236 0.2763 0.05136673088

item6step2 Gwet_AC2 0.77186 0.17009 0.001904500295

**BENCHMARKING**

**GPT-4-turbo**

item1step1

Landis-Koch CumProb

(0.8 to 1) Almost Perfect 0.63651

(0.6 to 0.8) Substantial 0.97814

(0.4 to 0.6) Moderate 0.99986

(0.2 to 0.4) Fair 1

(0 to 0.2) Slight 1

(-1 to 0) Poor 1

item1step2

Landis-Koch CumProb

(0.8 to 1) Almost Perfect 0.63651

(0.6 to 0.8) Substantial 0.97814

(0.4 to 0.6) Moderate 0.99986

(0.2 to 0.4) Fair 1

(0 to 0.2) Slight 1

(-1 to 0) Poor 1

item2step1

Landis-Koch CumProb

(0.8 to 1) Almost Perfect 0.13437

(0.6 to 0.8) Substantial 0.35143

(0.4 to 0.6) Moderate 0.60187

(0.2 to 0.4) Fair 0.80827

(0 to 0.2) Slight 0.92976

(-1 to 0) Poor 1

item2step2

Landis-Koch CumProb

(0.8 to 1) Almost Perfect 0.13437

(0.6 to 0.8) Substantial 0.35143

(0.4 to 0.6) Moderate 0.60187

(0.2 to 0.4) Fair 0.80827

(0 to 0.2) Slight 0.92976

(-1 to 0) Poor 1

item3step1

Landis-Koch CumProb

(0.8 to 1) Almost Perfect 0.11977

(0.6 to 0.8) Substantial 0.35047

(0.4 to 0.6) Moderate 0.63244

(0.2 to 0.4) Fair 0.85117

(0 to 0.2) Slight 0.95884

(-1 to 0) Poor 1

item3step2

Landis-Koch CumProb

(0.8 to 1) Almost Perfect 0.08987

(0.6 to 0.8) Substantial 0.26758

(0.4 to 0.6) Moderate 0.5118

(0.2 to 0.4) Fair 0.74502

(0 to 0.2) Slight 0.89981

(-1 to 0) Poor 1

item4step1

Landis-Koch CumProb

(0.8 to 1) Almost Perfect 0.05164

(0.6 to 0.8) Substantial 0.17519

(0.4 to 0.6) Moderate 0.38205

(0.2 to 0.4) Fair 0.62459

(0 to 0.2) Slight 0.82368

(-1 to 0) Poor 1

item4step2

Landis-Koch CumProb

(0.8 to 1) Almost Perfect 0.04084

(0.6 to 0.8) Substantial 0.2184

(0.4 to 0.6) Moderate 0.56286

(0.2 to 0.4) Fair 0.86203

(0 to 0.2) Slight 0.97829

(-1 to 0) Poor 1

item5step1

Landis-Koch CumProb

(0.8 to 1) Almost Perfect 0.46466

(0.6 to 0.8) Substantial 0.89192

(0.4 to 0.6) Moderate 0.9938

(0.2 to 0.4) Fair 0.99991

(0 to 0.2) Slight 1

(-1 to 0) Poor 1

item5step2

Landis-Koch CumProb

(0.8 to 1) Almost Perfect 0.22226

(0.6 to 0.8) Substantial 0.54944

(0.4 to 0.6) Moderate 0.82484

(0.2 to 0.4) Fair 0.95736

(0 to 0.2) Slight 0.99376

(-1 to 0) Poor 1

item6step2
 Landis-Koch CumProb

(0.8 to 1) Almost Perfect 0.37841

(0.6 to 0.8) Substantial 0.82842

(0.4 to 0.6) Moderate 0.98418

(0.2 to 0.4) Fair 0.99958

(0 to 0.2) Slight 1

(-1 to 0) Poor 1

**Gemini 2.5 Pro Preview**

item1step1

Landis-Koch CumProb

(0.8 to 1) Almost Perfect 0.94094

(0.6 to 0.8) Substantial 1

(0.4 to 0.6) Moderate 1

(0.2 to 0.4) Fair 1

(0 to 0.2) Slight 1

(-1 to 0) Poor 1

item1step2

Landis-Koch CumProb

(0.8 to 1) Almost Perfect 0.94094

(0.6 to 0.8) Substantial 1

(0.4 to 0.6) Moderate 1

(0.2 to 0.4) Fair 1

(0 to 0.2) Slight 1

(-1 to 0) Poor 1

item2step1

Landis-Koch CumProb

(0.8 to 1) Almost Perfect 0.17337

(0.6 to 0.8) Substantial 0.42552

(0.4 to 0.6) Moderate 0.68257

(0.2 to 0.4) Fair 0.86625

(0 to 0.2) Slight 0.95824

(-1 to 0) Poor 1

item2step2

Landis-Koch CumProb

(0.8 to 1) Almost Perfect 0.17337

(0.6 to 0.8) Substantial 0.42552

(0.4 to 0.6) Moderate 0.68257

(0.2 to 0.4) Fair 0.86625

(0 to 0.2) Slight 0.95824

(-1 to 0) Poor 1

item3step1

Landis-Koch CumProb

(0.8 to 1) Almost Perfect 0.41258

(0.6 to 0.8) Substantial 0.97461

(0.4 to 0.6) Moderate 0.99998

(0.2 to 0.4) Fair 1

(0 to 0.2) Slight 1

(-1 to 0) Poor 1

item3step2
 Landis-Koch CumProb

(0.8 to 1) Almost Perfect 0.09001

(0.6 to 0.8) Substantial 0.39231

(0.4 to 0.6) Moderate 0.77728

(0.2 to 0.4) Fair 0.96358

(0 to 0.2) Slight 0.99762

(-1 to 0) Poor 1

item4step1

Landis-Koch CumProb

(0.8 to 1) Almost Perfect 0.13308

(0.6 to 0.8) Substantial 0.37177

(0.4 to 0.6) Moderate 0.64922

(0.2 to 0.4) Fair 0.85821

(0 to 0.2) Slight 0.96021

(-1 to 0) Poor 1

item4step2

Landis-Koch CumProb

(0.8 to 1) Almost Perfect 0.08216

(0.6 to 0.8) Substantial 0.35

(0.4 to 0.6) Moderate 0.72085

(0.2 to 0.4) Fair 0.93937

(0 to 0.2) Slight 0.99399

(-1 to 0) Poor 1

item5step1

Landis-Koch CumProb

(0.8 to 1) Almost Perfect 0.51777

(0.6 to 0.8) Substantial 0.95174

(0.4 to 0.6) Moderate 0.9994

(0.2 to 0.4) Fair 1

(0 to 0.2) Slight 1

(-1 to 0) Poor 1

item5step2

Landis-Koch CumProb

(0.8 to 1) Almost Perfect 0.38539

(0.6 to 0.8) Substantial 0.88816

(0.4 to 0.6) Moderate 0.99643

(0.2 to 0.4) Fair 0.99998

(0 to 0.2) Slight 1

(-1 to 0) Poor 1

item6step2

Landis-Koch CumProb

(0.8 to 1) Almost Perfect 0.46176

(0.6 to 0.8) Substantial 0.88785

(0.4 to 0.6) Moderate 0.99312

(0.2 to 0.4) Fair 0.99989

(0 to 0.2) Slight 1

(-1 to 0) Poor 1

**DeepSeek-R1**

item1step1

Landis-Koch CumProb

(0.8 to 1) Almost Perfect 0.97356

(0.6 to 0.8) Substantial 1

(0.4 to 0.6) Moderate 1

(0.2 to 0.4) Fair 1

(0 to 0.2) Slight 1

(-1 to 0) Poor 1

item1step2

Landis-Koch CumProb

(0.8 to 1) Almost Perfect 0.97356

(0.6 to 0.8) Substantial 1

(0.4 to 0.6) Moderate 1

(0.2 to 0.4) Fair 1

(0 to 0.2) Slight 1

(-1 to 0) Poor 1

item2step1

Landis-Koch CumProb

(0.8 to 1) Almost Perfect 0.11977

(0.6 to 0.8) Substantial 0.32504

(0.4 to 0.6) Moderate 0.57454

(0.2 to 0.4) Fair 0.78961

(0 to 0.2) Slight 0.92106

(-1 to 0) Poor 1

item2step2

Landis-Koch CumProb

(0.8 to 1) Almost Perfect 0.11977

(0.6 to 0.8) Substantial 0.32504

(0.4 to 0.6) Moderate 0.57454

(0.2 to 0.4) Fair 0.78961

(0 to 0.2) Slight 0.92106

(-1 to 0) Poor 1

item3step1

Landis-Koch CumProb

(0.8 to 1) Almost Perfect 0.02801

(0.6 to 0.8) Substantial 0.10895

(0.4 to 0.6) Moderate 0.27252

(0.2 to 0.4) Fair 0.50372

(0 to 0.2) Slight 0.73233

(-1 to 0) Poor 1

item3step2

Landis-Koch CumProb

(0.8 to 1) Almost Perfect 0.00059

(0.6 to 0.8) Substantial 0.01993

(0.4 to 0.6) Moderate 0.19208

(0.2 to 0.4) Fair 0.62348

(0 to 0.2) Slight 0.93313

(-1 to 0) Poor 1

item4step1

Landis-Koch CumProb

(0.8 to 1) Almost Perfect 0.02242

(0.6 to 0.8) Substantial 0.07267

(0.4 to 0.6) Moderate 0.16523

(0.2 to 0.4) Fair 0.30541

(0 to 0.2) Slight 0.47995

(-1 to 0) Poor 1

item4step2

Landis-Koch CumProb

(0.8 to 1) Almost Perfect 0.00118

(0.6 to 0.8) Substantial 0.04645

(0.4 to 0.6) Moderate 0.37396

(0.2 to 0.4) Fair 0.85025

(0 to 0.2) Slight 0.99172

(-1 to 0) Poor 1

item5step1

Landis-Koch CumProb

(0.8 to 1) Almost Perfect 0.13529

(0.6 to 0.8) Substantial 0.36589

(0.4 to 0.6) Moderate 0.63192

(0.2 to 0.4) Fair 0.83968

(0 to 0.2) Slight 0.94949

(-1 to 0) Poor 1

item5step2

Landis-Koch CumProb

(0.8 to 1) Almost Perfect 0.0718

(0.6 to 0.8) Substantial 0.20997

(0.4 to 0.6) Moderate 0.41041

(0.2 to 0.4) Fair 0.62963

(0 to 0.2) Slight 0.8104

(-1 to 0) Poor 1

item6step2

Landis-Koch CumProb

(0.8 to 1) Almost Perfect 0.37841

(0.6 to 0.8) Substantial 0.82842

(0.4 to 0.6) Moderate 0.98418

(0.2 to 0.4) Fair 0.99958

(0 to 0.2) Slight 1

(-1 to 0) Poor 1

**Qwen3-235B-A22B**

item1step1

Landis-Koch CumProb

(0.8 to 1) Almost Perfect 0.94094

(0.6 to 0.8) Substantial 1

(0.4 to 0.6) Moderate 1

(0.2 to 0.4) Fair 1

(0 to 0.2) Slight 1

(-1 to 0) Poor 1

item1step2

Landis-Koch CumProb

(0.8 to 1) Almost Perfect 0.94094

(0.6 to 0.8) Substantial 1

(0.4 to 0.6) Moderate 1

(0.2 to 0.4) Fair 1

(0 to 0.2) Slight 1

(-1 to 0) Poor 1

item2step1

Landis-Koch CumProb

(0.8 to 1) Almost Perfect 0.11049

(0.6 to 0.8) Substantial 0.30819

(0.4 to 0.6) Moderate 0.5572

(0.2 to 0.4) Fair 0.77803

(0 to 0.2) Slight 0.9159

(-1 to 0) Poor 1

item2step2

Landis-Koch CumProb

(0.8 to 1) Almost Perfect 0.11049

(0.6 to 0.8) Substantial 0.30819

(0.4 to 0.6) Moderate 0.5572

(0.2 to 0.4) Fair 0.77803

(0 to 0.2) Slight 0.9159

(-1 to 0) Poor 1

item3step1

Landis-Koch CumProb

(0.8 to 1) Almost Perfect 0.10862

(0.6 to 0.8) Substantial 0.32289

(0.4 to 0.6) Moderate 0.59691

(0.2 to 0.4) Fair 0.82414

(0 to 0.2) Slight 0.9463

(-1 to 0) Poor 1

item3step2

Landis-Koch CumProb

(0.8 to 1) Almost Perfect 0.22364

(0.6 to 0.8) Substantial 0.55106

(0.4 to 0.6) Moderate 0.82566

(0.2 to 0.4) Fair 0.95755

(0 to 0.2) Slight 0.99378

(-1 to 0) Poor 1

item4step1

Landis-Koch CumProb

(0.8 to 1) Almost Perfect 0.01933

(0.6 to 0.8) Substantial 0.07016

(0.4 to 0.6) Moderate 0.17382

(0.2 to 0.4) Fair 0.33774

(0 to 0.2) Slight 0.53878

(-1 to 0) Poor 1

item4step2

Landis-Koch CumProb

(0.8 to 1) Almost Perfect 0.09157

(0.6 to 0.8) Substantial 0.49906

(0.4 to 0.6) Moderate 0.90507

(0.2 to 0.4) Fair 0.99563

(0 to 0.2) Slight 0.99996

(-1 to 0) Poor 1

item5step1

Landis-Koch CumProb

(0.8 to 1) Almost Perfect 0.15156

(0.6 to 0.8) Substantial 0.40129

(0.4 to 0.6) Moderate 0.67349

(0.2 to 0.4) Fair 0.86978

(0 to 0.2) Slight 0.9634

(-1 to 0) Poor 1

item5step2

Landis-Koch CumProb

(0.8 to 1) Almost Perfect 0.19855

(0.6 to 0.8) Substantial 0.50086

(0.4 to 0.6) Moderate 0.77962

(0.2 to 0.4) Fair 0.93525

(0 to 0.2) Slight 0.98784

(-1 to 0) Poor 1

item6step2

Landis-Koch CumProb

(0.8 to 1) Almost Perfect 0.37841

(0.6 to 0.8) Substantial 0.82842

(0.4 to 0.6) Moderate 0.98418

(0.2 to 0.4) Fair 0.99958

(0 to 0.2) Slight 1

(-1 to 0) Poor 1
