# Supplementary material for: Assessing the risk of bias of clinical trials with large language models and ROBUST-RCT: a feasibility study
Source: Sci Rep. 2026 Mar 17;16:13723. doi: 10.1038/s41598-026-44303-z (PMC13125330; doi:10.1038/s41598-026-44303-z)
Supplement: Supplementary file 5 — Supplementary Information 5. [file 41598_2026_44303_MOESM5_ESM.docx]

**Supplementary Table 1.** Pre-specified exclusion criteria.

| **Reason** | **Description** |
| --- | --- |
| 1. Not an RCT | Studies that are not randomized controlled trials (e.g., single-arm studies, systematic reviews, case reports, poster presentations, randomized controlled trial protocol without results). |
| 2. Not individually randomized | Studies that are not individually randomized human trials (e.g., cluster-randomized trials, crossover designs, animal studies). |
| 3. Not the original study | Post-hoc analyses and secondary analyses that were not the original primary purpose and main outcome of the study design, even if planned before the trial. |
| 4. Other | Other studies for which ROBUST-RCT analysis is not feasible or useful (e.g., lack of access to the full article, language isn’t English). |
