# Supplementary material for: Assessing the risk of bias of clinical trials with large language models and ROBUST-RCT: a feasibility study
Source: Sci Rep. 2026 Mar 17;16:13723. doi: 10.1038/s41598-026-44303-z (PMC13125330; doi:10.1038/s41598-026-44303-z)
Supplement: Supplementary file 7 — Supplementary Information 7. [file 41598_2026_44303_MOESM7_ESM.docx]

**Supplementary Table 3.** Pre-specified thresholds for item 6 of ROBUST-RCT.

| Definitely low | Missing data < 5% |
| --- | --- |
| Probably low | 5% ≤ Missing data < 10% |
| Probably high | 10% ≤ Missing data < 15% |
| Definitely high | Missing data ≥ 15% |
