# Supplementary material for: Assessing the risk of bias of clinical trials with large language models and ROBUST-RCT: a feasibility study
Source: Sci Rep. 2026 Mar 17;16:13723. doi: 10.1038/s41598-026-44303-z (PMC13125330; doi:10.1038/s41598-026-44303-z)
Supplement: Supplementary file 9 — Supplementary Information 9. [file 41598_2026_44303_MOESM9_ESM.docx]

**Supplementary Table 5.** Benchmark with cumulative probabilities of results in each Landis and Koch classification by Gwet's method.

| **Landis and Koch** | **Human consensus and GPT-4-turbo** | **Human consensus and Gemini 2.5 Pro Preview** | **Human consensus and DeepSeek-R1** | **Human consensus and Qwen3-235B-A22B** |
| --- | --- | --- | --- | --- |
| 0.8 to 1  (almost perfect) | 0.02719 | 0.07586 | 0.00115 | 0.06934 |
| 0.6 to 0.8  (substantial) | 0.48904 | 0.88868 | 0.1069 | 0.70438 |
| 0.4 to 0.6  (moderate) | **0.96912** | **0.99995** | 0.71251 | **0.99467** |
| 0.2 to 0.4  (fair) | 0.99992 | 1 | **0.99098** | 1 |
| 0.0 to 0.2  (slight) | 1 | 1 | 0.99998 | 1 |
| -1 to 0 (poor) | 1 | 1 | 1 | 1 |
