# Supplementary material for: Assessing the risk of bias of clinical trials with large language models and ROBUST-RCT: a feasibility study
Source: Sci Rep. 2026 Mar 17;16:13723. doi: 10.1038/s41598-026-44303-z (PMC13125330; doi:10.1038/s41598-026-44303-z)
Supplement: Supplementary file 10 — Supplementary Information 10. [file 41598_2026_44303_MOESM10_ESM.docx]

**Supplementary Table 6.** Fleiss’ Kappa among the three reviewers (before consensus).

| **Fleiss’ Kappa** | **Percentage of Agreement** | **Percentage Expected** | **Standard Error** | **95% Confidence Interval** |
| --- | --- | --- | --- | --- |
| 0.4941 | 0.6666 | 0.3410 | 0.0947 | 0.304, 0.684 |
