# Supplementary material for: Assessing the risk of bias of clinical trials with large language models and ROBUST-RCT: a feasibility study
Source: Sci Rep. 2026 Mar 17;16:13723. doi: 10.1038/s41598-026-44303-z (PMC13125330; doi:10.1038/s41598-026-44303-z)
Supplement: Supplementary file 11 — Supplementary Information 11. [file 41598_2026_44303_MOESM11_ESM.docx]

**Supplementary Table 7.** Mean ordinal values for each group. The assigned values ranged from 0 (definitely yes, definitely low) to 3 (definitely no, definitely high); thus, lower values indicate a more lenient assessment, whereas higher values indicate a stricter assessment.

| **Reviewer** | **Mean** | **Standard Deviation** |
| --- | --- | --- |
| Human consensus | 0.81 | 1.00 |
| GPT-4-turbo | 0.99 | 1.15 |
| Gemini 2.5 Pro Preview | 0.76 | 0.91 |
| DeepSeek-R1 | 1.16 | 1.07 |
| Qwen3-235B-A22B | 0.86 | 1.12 |
