# Supplementary material for: Assessing the risk of bias of clinical trials with large language models and ROBUST-RCT: a feasibility study
Source: Sci Rep. 2026 Mar 17;16:13723. doi: 10.1038/s41598-026-44303-z (PMC13125330; doi:10.1038/s41598-026-44303-z)
Supplement: Supplementary file 12 — Supplementary Information 12. [file 41598_2026_44303_MOESM12_ESM.docx]

**Supplementary Table 8.** Direction of bias analysis. Wilcoxon Signed-Rank test. DeepSeek had the two endpoints of the confidence interval with negative values, suggesting it's a more stringent reviewer than the human consensus. The results were rounded to two decimal places.

| **Comparison** | **Median** | **95% CI** | **p-value** | **adjusted p-value (Bonferroni)** |
| --- | --- | --- | --- | --- |
| GPT-4-turbo | -0.50 | -1.00, 0.00 | 0.11 | 0.43 |
| Gemini 2.5 Pro Preview | 0.00 | -0.00, 1.00 | 0.65 | 1 |
| DeepSeek-R1 | -1.00 | -1.00, 0.00 | **< 0.01*** | **0.02*** |
| Qwen3-235B-A22B | 0.00 | -1.00, 0.50 | 0.72 | 1 |
| ***Significance** | | | | |
